# Modulation of cerebellar cortical, cerebellar nuclear and vestibular nuclear activity using alternating electric currents

**DOI:** 10.3389/fnsys.2023.1173738

**Published:** 2023-05-18

**Authors:** Billur Avlar, Ramia Rahman, Sai Vendidandi, Esma Cetinkaya, Ahmet S. Asan, Mesut Sahin, Eric J. Lang

**Affiliations:** ^1^Department of Neuroscience and Physiology, NYU Neuroscience Institute, New York University Grossman School of Medicine,, New York, NY, United States; ^2^Department of Biomedical Engineering, New Jersey Institute of Technology, Newark, NJ, United States

**Keywords:** transcranial stimulation, olivocerebellar system, cerebellar nuclei, Purkinje cell, electric fields in neural tissue

## Abstract

**Introduction:**

Cerebellar transcranial alternating current stimulation (ctACS) has shown promise as a therapeutic modality for treating a variety of neurological disorders, and for affecting normal learning processes. Yet, little is known about how electric fields induced by applied currents affect cerebellar activity in the mammalian cerebellum under *in vivo* conditions.

**Methods:**

Alternating current (AC) stimulation with frequencies from 0.5 to 20 Hz was applied to the surface of the cerebellum in anesthetized rats. Extracellular recordings were obtained from Purkinje cells (PC), cerebellar and vestibular nuclear neurons, and other cerebellar cortical neurons.

**Results and discussion:**

AC stimulation modulated the activity of all classes of neurons. Cerebellar and vestibular nuclear neurons most often showed increased spike activity during the negative phase of the AC stimulation. Purkinje cell simple spike activity was also increased during the negative phase at most locations, except for the cortex directly below the stimulus electrode, where activity was most often increased during the positive phase of the AC cycle. Other cortical neurons showed a more mixed, generally weaker pattern of modulation. The patterns of Purkinje cell responses suggest that AC stimulation induces a complex electrical field with changes in amplitude and orientation between local regions that may reflect the folding of the cerebellar cortex. Direct measurements of the induced electric field show that it deviates significantly from the theoretically predicted radial field for an isotropic, homogeneous medium, in both its orientation and magnitude. These results have relevance for models of the electric field induced in the cerebellum by AC stimulation.

## Introduction

Recent research provides evidence supporting the use of cerebellar transcranial electrical stimulation (ctES) as a potential treatment for a variety of brain disorders. For example, ctES has been reported to improve motor learning, and cognitive and emotional processes in normal and brain injured individuals (Ferrucci et al., 2012; Naro et al., 2017a,b; Darch et al., 2018). Yet, relatively little information exists on how ctES, and more generally, applied electric fields, alter cerebellar activity. In particular, animal studies on transcranial AC stimulation (tACS) are almost non-existent in the literature. Such information could provide insight toward improving the clinical efficacy and reliability of ctES, and increase the potential of applied electric fields as a tool for probing cerebellar function.

Much of what is known about how electric fields modulate cerebellar activity comes from a classic set of studies by Chan and Nicholson (1986) and Chan et al. (1988) who studied the relationship between electric field orientation and the modulation of PC activity in an *in vitro* turtle cerebellum preparation. The flat nature of the turtle cerebellum and the *in vitro* design that was used allowed for AC stimulation to produce a spatially uniform electric field throughout the cerebellum, which, in turn, allowed the dependence of simple spike (SS) responses on the relative orientation of the electric field and PC somatodendritic axis to be determined. AC stimulation that generated an electric field oriented in the pial-ventricular axis (i.e., oriented parallel to the main somatodendritic axis of the PC) was found to strongly modulate PC activity. Moreover, the vast majority of PCs whose activity was modulated showed increased SS activity when the field was directed to cause current flow from distal dendrites toward the soma (somatopetally), and conversely, decreased activity when the field direction was reversed, in line with studies on other brain regions (Purpura and McMurtry, 1965; Ranck, 1975; Bikson et al., 2004; Rahman et al., 2013).

How these results translate to the more complex situation presented by the mammalian cerebellum *in vivo* remains to be determined. In our previous study, we reported SS responses to AC stimulation for PCs located directly subjacent to the stimulus electrode. These responses were found to be largely consistent with the Chan and Nicholson findings with regard to SS responses; that is, most, but not all, PCs increased their SS activity during the positive phase of the AC cycle (when current was flowing inward from the local pial surface) (Asan et al., 2020). However, the folded nature of the mammalian cerebellum raises the question of whether a more complex pattern of current flow may exist in it, particularly under *in vivo* conditions, one which could result in different response patterns for PCs located in regions of the cerebellar cortex that are farther from the stimulation site.

PCs form the sole output of the cerebellar cortex and target both the cerebellar nuclei (CN) and vestibular nuclei (VN). Thus, modulation of PC activity by AC stimulation suggests that the activity of CN and VN cells is indirectly modulated. AC stimulation could also influence CN and VN activity directly, if the electric field remains strong enough within these nuclei despite their distance from the stimulating electrode. As these nuclei mediate the cerebellum’s influence on other brain areas, it is of interest to know how AC stimulation modulates their activity, because this information could help optimize use of the cerebellum as a therapeutic tool.

To address the above issues and provide a more complete characterization of how AC stimulation alters cerebellar activity, extracellular recordings during AC stimulation of the cerebellum were obtained from PCs, other cortical cells, CN cells, and VN cells.

## Materials and methods

Experiments were performed in accordance with the National Institute of Health *Guide for the Care and Use of Laboratory Animals*. Experimental protocols were approved by the Institutional Animal Care and Use Committee of New York University Grossman School of Medicine or that of Rutgers University, depending on which location an experiment was performed. The bulk of the recordings, 61/71 cells, were obtained at NYU and the surgical procedures below relate to these recordings. The remaining recordings were obtained on Rutgers-Newark Campus through an agreement with New Jersey Institute of Technology, and the procedures for recording those cells are described in a prior publication (Asan et al., 2020).

### Surgical procedures

Surgeries were performed on adult male and female Sprague-Dawley and Long-Evans rats (*n* = 19). Anesthesia was induced by an injection of ketamine (100 mg/kg) and xylazine (8 mg/kg) intraperitoneally. After the animal reached a surgical plane of anesthesia, a tracheotomy was performed to allow mechanical ventilation, if needed, and a femoral vein catheter was inserted in some animals. In animals with a femoral vein catheter, supplemental anesthesia was given as a continuous intravenous injection of a ketamine-xylazine mixture (ketamine, 6 μg/kg/min; xylazine, 1 μg/kg/min). In the remaining animals, additional doses of ketamine were given intraperitoneally, as needed. The depth of anesthesia was maintained such that pedal reflexes were absent throughout the experiment through perfusion at the conclusion of the recording session. Temperature was measured by a rectal probe and maintained at 37°C using a heating pad (TR-200, Fine Science Tools, Foster City, CA, USA). Animals were placed in a stereotaxic frame and a craniotomy was performed to expose most of the dorsal surface of the posterior lobe of the cerebellum. Specifically, the bone overlying crus1 and caudally to the foramen magnum was removed from roughly the entire vermis and from the hemisphere on one side to the point at which the cerebellar surface turns sharply in the ventral direction. The dura mater was incised to create an opening for inserting the recording microelectrode, and a stimulation electrode was placed on the cerebellar surface. The dura was then covered with saline soaked gelfoam or a layer of agar, except for the entry point of the recording electrode and the region under the stimulation electrode.

### Recording and stimulation procedures

For experiments in which single unit responses to AC stimulation were recorded, the stimulation electrode was constructed from a chlorided silver wire (diameter 0.015″, A-M Systems, #7830, Sequim, WA, USA). The end of the wire that was placed on the brain was shaped into a 3 mm diameter, 2–3 turn coil. The base of the coil was placed on the cortex of vermal lobule 6 and/or on the medial aspect of the crus 1. The indifferent electrode was a silver chlorided wire that was placed in the shoulder region in the space between the muscles and overlying skin.

Following placement of the stimulation electrode, extracellular spike recordings were made with a NaCl (1 M)-filled glass microelectrode. Electrodes were inserted into the cerebellum using a joy-stick controlled micromanipulator (Burleigh Inchworm LSS-1000) to search for cells. The electrode was inserted caudal to the stimulation electrode, either through crus 2 or the paramedian lobule, angled to target the CN. Recordings were obtained from cells in the cortex, CN, and VN. Recordings were amplified with either a Dagan amplifier (Model EX1, 500–1,000× gain, filtered at 300 Hz and 10 kHz) or A-M systems AC amplifier (Model 1700 with 1,000× gain and filtered at 300 Hz and 10 kHz) and recorded using a multichannel recording system (MultiChannel Systems MCS GmbH, Reutlingen, Germany) with a sampling rate of 25 kHz/channel.

When a single unit was isolated, spontaneous activity was generally recorded for an initial 3-min period followed by 3-min periods in which AC stimulation at a particular frequency was interleaved with periods of non-stimulation (10 s on/10 s off). A final period of spontaneous activity was recorded when possible. AC stimuli were generated by an isolated current stimulator (Model SYS-A395D, WPI, Sarasota, FL, USA) driven by a waveform generator (Model 185, Wavetek, San Diego, CA, USA). The following nominal frequencies were tested for most cells: 0.5 Hz, 2 Hz, 5 Hz, and 20 Hz. Because the waveform generator had an analog frequency selector knob, stimulation frequency varied slightly from the nominal frequencies across recordings. Overall, the range of actual stimulation frequencies for each nominal frequency was: 0.5 Hz, 0.48–0.51; 2 Hz, 1.84–1.94; 5 Hz, 4.84–5.06; and 20 Hz, 18.64–19.31. For simplicity, in the text and figure axis labels, the nominal frequencies are listed, but for statistical analyses in which stimulation frequency was an independent variable, the exact stimulation frequency was used. Peak stimulus amplitude was 500 μA for the majority of the new recordings (36/61). In the remaining recordings amplitudes of 600 (10), 700 (9), 800 (1), 900 (1), or 1,000 (4) μA were used because modulation was not evident with 500 μA. Recordings at Rutgers used intensities ranging between 100–250 μA.

For experiments in which the electric field was measured, the stimulation electrode was constructed from a silver wire (diameter 0.015″, A-M Systems, #7830) that was bent into an “L” shape, with the foot of the L having a length of ∼2 mm. The foot of the L was placed on the surface of the vermis (lobule 6 or 7) such that it ran in the mediolateral direction (i.e., longitudinally within a lobule). Immediately caudal to the electrode, a silicon rubber platform with an electron microscopic grid on its outer surface was cemented in place (Lang, 2018). The platform was positioned such that the grid rows and columns were oriented mediolaterally and rostrocaudally. The center-to-center spacing of the grid holes was 254 μm. To record the fields induced by stimuli, an electrode was inserted through holes in the grid and then lowered through the cerebellum perpendicular to the cerebellar surface at the entry point. Responses to current pulses were recorded at 200-μm intervals along each track to a depth of 4 mm. Successive tracks were generally made at ∼254-μm distances using the spacings of the grid holes as a guide.

### Data analysis

Data files containing the extracellular recording traces, AC stimulus wave, and trigger times marking the start of the stimulation periods were converted to general binary or text files using MC_Datatool software (MultiChannel Systems MCS GmbH) and then imported into Igor Pro 8 (Wavemetrics, Lake Oswego, OR, USA). Analyses were performed using built-in and custom-written routines with Igor Pro and Microsoft Excel for Mac.

To eliminate artifacts due to the AC stimulus, recordings were high-pass filtered using a finite impulse response (FIR) filter with a Hanning window and 150 Hz cut-off (number of coefficients 701). Potential spikes were detected by either a single voltage threshold or two (upper and lower) thresholds with a time limit of 1–2 ms for crossing both thresholds. Next, principal components analysis (PCA) was performed on the detected spike waveforms, and a cluster plot of the first two principal components were used to isolate spikes from individual cells.

#### Calculation of circular mean of spike activity during AC stimulation

Each spike during stimulation was represented as a unit vector in the direction equal to its phase with respect to the AC cycle. These unit vectors were then added, and the magnitude of the resultant vector was divided by the total number of unit vectors to obtain the circular mean vector. The direction of the circular mean vector provides an overall measure of the phase of the spike activity, and the magnitude, which can range from 0 to 1, gives a measure of the strength of the modulation.

#### Calculation of population vectors

To provide an overall measure of the response to AC stimulation by a particular cell type, a population vector was calculated from the individual cell circular mean vectors by summing them and dividing the resultant by the number of cells in the sum.

#### Calculation of electric field components in the parasagittal plane from experimental data

The responses to positive current pulses delivered via the stimulating electrode were used to calculate the dorsoventral and rostrocaudal components of the electric field at different locations in the brain. The dorsoventral component was computed as the difference in the voltage response to a current pulse at two successive locations (superficial minus deep) along the same track divided by the difference in depth (200 μm). The rostrocaudal component was determined from the difference of responses at the same depth on neighboring tracks that were aligned in the same parasagittal plane but were separated by a fixed distance in the rostral caudal axis (rostral minus caudal) divided by the intertrack spacing (multiple of 254 μm). The direction of the field was then computed as arctan (θ), where tan (θ) is equal to the ratio of the dorsoventral to the rostrocaudal component. The coordinates (dorsoventral and rostrocaudal) of the field were taken as the midpoints of the two measurement locations used to calculate the respective components.

### Histological and stereotaxic procedures for identifying cell locations

Following the completion of each recording track, alcian blue dye was injected at the depths where cell activity was recorded and/or at the end of the track. At the conclusion of the experiment, the animal was perfused with normal saline followed by 10% formalin. The brain was removed, soaked in 10% formalin for at least 1–2 days, and then transferred to a 30% sucrose/10% formalin solution until it sank. Parasagittal 60-μm sections of the cerebellum were prepared on a freezing microtome and mounted on chrome–alum gelatin-coated slides for counterstaining with cresyl violet. To identify cell locations, tissue shrinkage was calculated by measuring the distance from the brain surface at the electrode entry point to the dye spot and taking the ratio of this measurement to the micrometer value of the dye spot. Micrometer values for the depths of recorded cells were then reduced by this factor and then plotted along the track. Typically, tissue shrinkage was ∼10%.

In experiments where multiple tracks were made, dye spots were only made on some tracks to avoid ambiguity in defining each track. In these cases, cells could be recorded on unmarked tracks, and their location was defined using the micrometer readings and comparison to the nearest dye-labeled track. In addition, cells recorded at depths less than < 3.0 mm were categorized as cortical neurons, as the shortest distance from the surface of the crus 2 or paramedian lobules to the most superficial portion of the cerebellar nuclei is ∼3.0 mm.

### Statistics

Unless stated otherwise, *t*-tests, paired or unpaired, or ANOVA, as appropriate, were used to test for statistical significance. Other statistical tests were either based on the built-in functions in Igor Pro 8, R software (4.1.0), or are from formulas given in Zar (1999). These include: correlation significance (Pearson’s r ≠ 0) (Zar), Rayleigh and Moore’s version of Rayleigh tests for uniformity of a circular distribution (Igor), Kolmogorov–Smirnov (KS) goodness-of-fit (Igor). In the text, mean values are presented with their standard deviation.

## Results

### Database

**FIGURE 1:**
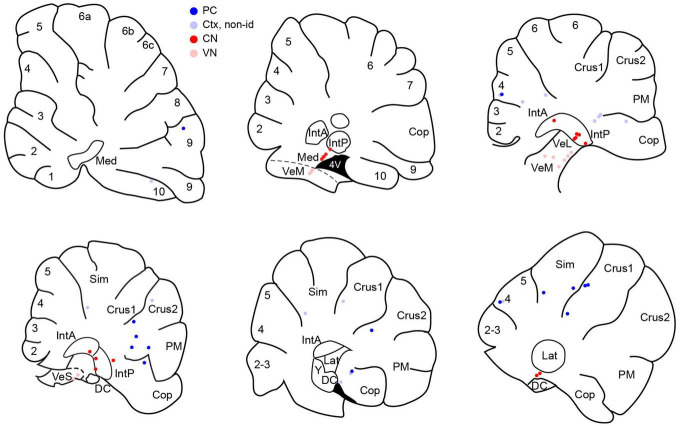
Distribution of histologically-localized neurons. Schematics of parasagittal sections through the cerebellum at mediolateral distances going from approximately 0.5 to 3.5 mm from the midline. Dark blue circles indicate PCs. Pale blue circles are non-identified cortical neurons. Red circles indicate CN neurons and pink circles indicate VN neurons. Numbers on schematics correspond to lobule numbers. Cop, copula; DC, dorsal cochlear nucleus; IntA, IntP, anterior and posterior interpositus; Lat, lateral CN; Med, medial CN; PM, paramedian; Sim, simplex; VeL, VeM, VeS, lateral, medial, superior vestibular nucleus.

The following results are based on recordings from 71 neurons, including 61 newly recorded cells and 10 PCs that were included in the database of a prior paper and are here classified as belonging to the local PC group (defined below). Histological, stereotaxic, and physiological criteria were used to classify cells as CN (*n* = 20), VN (*n* = 11), or cerebellar cortical neurons (*n* = 40). Cortical neurons were further categorized as PCs (*n* = 25) and non-identified cortical cells (*n* = 15). To be classified as a PC, the SS had to show a CS-associated pause. PCs were further categorized according to their location with respect to the stimulus electrode, as either local PCs (located at or near the apex of the lobule on which the coil rested; *n* = 10) or distant PCs (all other PCs; *n* = 15). Figure 1 shows the distribution of histologically-identified cells, with the exception of the local PCs, which were located on the apex of vermal lobule 7.

### PC-SS responses to AC stimulation

Simple spikes were recorded from 15 PCs distant to the stimulation electrode. Their distribution across the cerebellar cortex is shown in Figure 1 (dark blue circles). These PCs displayed a range of spontaneous SS firing rates (average, 19.08 ± 8.84 Hz; range, 5.21–39.44 Hz), as calculated from the interleaved 10-s periods of spontaneous activity (a total of ∼ 1 min) separating the 0.5 Hz AC stimulation periods or from a 3-min period recorded just prior to starting AC stimulation.

**FIGURE 2:**
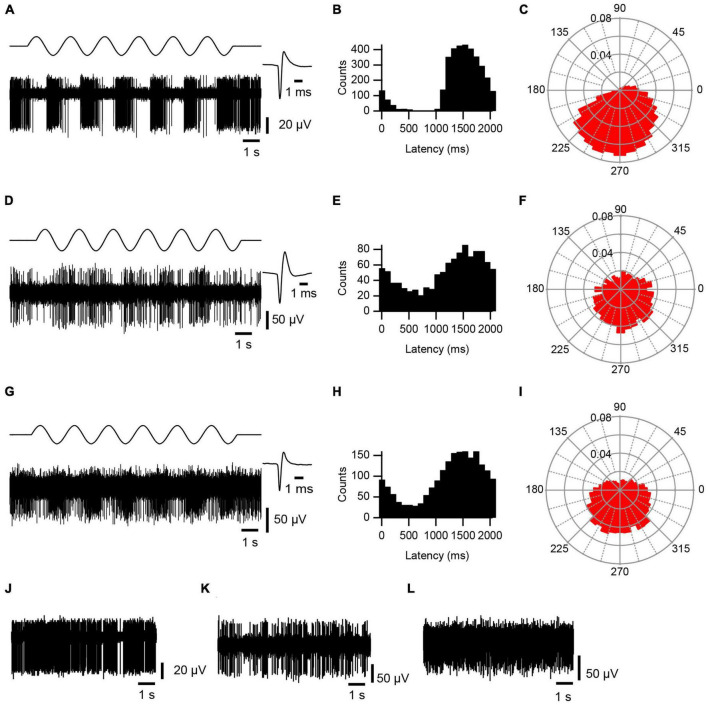
AC modulation of cerebellar activity. Responses of PC SS **(A–C)**, CN **(D–F)** and VN **(G–I)** activity to ∼0.5 Hz AC stimulation. **(A,D,G)** Bottom trace of each panel is an extracellular recording of PC, CN cell, and VN cell, respectively. Top trace shows AC stimulation. Average waveform for each cell is shown at an expanded time scale to the right (*n* = 500 spikes). **(B,E,H)** Histograms of spike activity triggered of off the start of each sinusoidal cycle. Histograms compiled from 6 to 9 stimulus trains, each consisting of 6 complete cycles. Bins were set to 1/20 of the cycle period (∼100 ms for ∼0.5 Hz). **(C,F,I)** Rose plots showing the phase distribution of activity relative to the start of the sinusoidal cycle. Radial axis plots fraction of spikes. Bin size is 10°. **(J–L)** Spontaneous activity of the PC **(J)**, CN **(K)**, and VN **(L)** cells shown in panels **(A,D,G)**, respectively.

**FIGURE 3:**
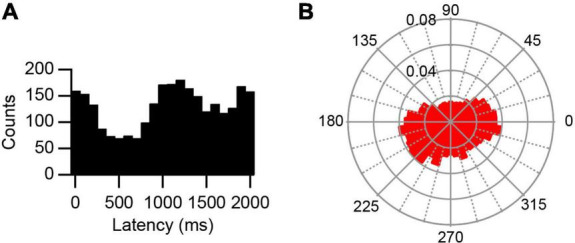
Bimodal response to AC stimulation. **(A)** Histogram of PC SS activity triggered off of the start of the sine wave cycle during 0.5 Hz stimulation. Note one peak shortly after 1,000 ms and one that spans the ends of the histogram. **(B)** Rose plot shows increased responses occurred between 345° – 30° and between 180° – 240°.

AC stimulation caused a strong phase-locked modulation of activity, as shown by the recording in Figure 2A, which was not present in PC spontaneous activity (Figure 2J). The modulation was first assessed by plotting the phase distribution of the SS activity relative to the start of the AC cycle on standard histograms and polar plots, where 0 ms or 0°, respectively, marks the start of the AC cycle (Figures 2B, C). By visual inspection, histograms and polar plots were classified as modulating (with uni- or bi-modal distributions) or non-modulating (flat or noisy). In the large majority of cases, AC stimulation produced a unimodal modulation of SS activity (0.5 Hz: 14/15 modulation, 13/14 unimodal distribution; 2 Hz: 11/14, 11/11; 5 Hz: 8/10, 8/8; 20 Hz: 6/6, 6/6). For comparison, an exceptional bimodal distribution in response to 0.5 Hz stimulation is shown in Figure 3, where one peak is present around 1,000–1,200 ms (∼180°–240°) and the other spans the beginning and end of the AC cycle (∼345°–30°).

**FIGURE 4:**
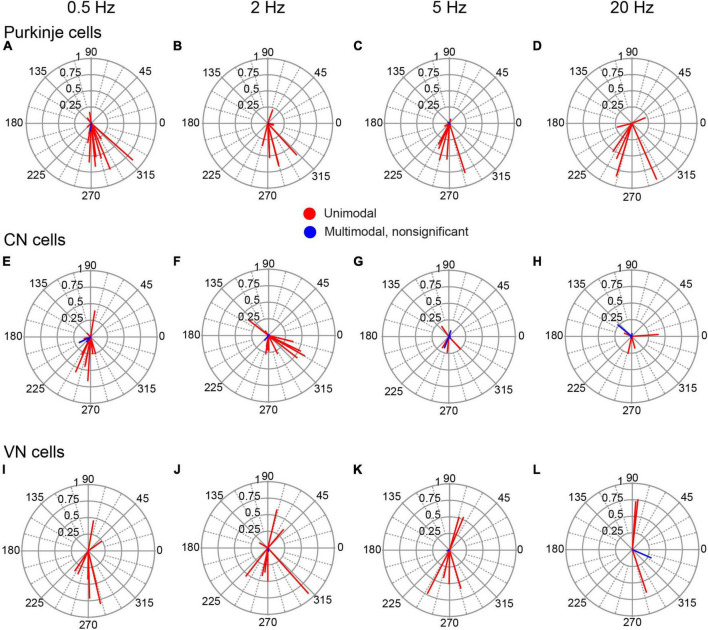
Phase distribution of PC, CN, and VN responses to AC stimulation. The individual cell circular means for activity during AC stimulation at different frequencies are shown for PCs **(A–D)**, CN cells **(E–H)** and VN cells **(I–L)**. Each column of plots shows the responses to the AC frequency indicated at the top of the column. In each plot, the lines represent the circular means for individual cells.

To quantify the modulation and test its statistical significance, we calculated the circular mean of the SS activity of each PC during each stimulation condition. These are shown as vectors for all PCs at each frequency in Figures 4A–D. Most of the circular means were highly statistically significant, confirming the visual categorization (Moore test; 0.5 Hz, *p* < 2 × 10^–4^, *n* = 14/15 cells; 2 Hz, *p* < 1 × 10^–7^, *n* = 11/14; 5 Hz, *p* < 7 × 10^–3^, *n* = 8/10; 20 Hz, *p* < 5 × 10^–26^, *n* = 6/6). Indeed, only one PC failed to show modulation at all tested frequencies. Although nearly all PC SS activity showed evidence of modulation, the amplitudes of their vectors varied widely (0.407 ± 0.291; range, 0.05 to 0.951; *n* = 32/38 conditions with significant vectors), with some having near maximal modulation (i.e., an amplitude close to one), indicating a strong phase-locking of activity. Of particular note, at each AC frequency, the large majority of PCs with significant cell vectors for unimodal distributions had phase angles between 180° and 360° (0.5 Hz, 10/13; 2 Hz, 10/11; 5 Hz, 7/8; 20 Hz, 5/6), which were generally close to 270°, indicating that SS activity modulated out-of-phase with respect to the AC stimulation.

**FIGURE 5:**
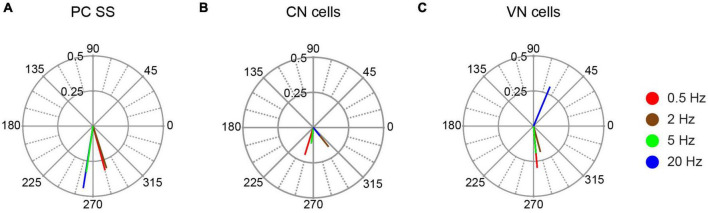
Population vector plots of responses to AC stimulation. The polar plots show the population vectors for responses of PC SS **(A)**, CN cells **(B)**, and VN cells **(C)** to AC stimulation at the tested stimulus frequencies. Population vectors were calculated from all cells with significant unimodal responses.

This out-of-phase relationship was further characterized by calculating the population vectors at each stimulation frequency (Figure 5A). All population vectors showed phase angles close to 270°, with the 0.5 and 2 Hz vectors having slightly larger angles, and the 5 and 20 Hz vectors having slightly smaller ones. The amplitudes of the vectors were similar, except for the 20 Hz vector, which was larger than the others.

We considered two possible explanations of the increased amplitude of the 20 Hz population vector: either stronger modulation of the individual PC SS trains or closer alignment of the phases across the PC population. To test for changes in the amplitude of the modulation of individual PCs, the average amplitude of the individual cell circular means was compared across frequencies: 0.5 Hz, 0.40 ± 0.28, *n* = 13; 2 Hz, 0.37 ± 0.23, *n* = 11; 5 Hz, 0.37 ± 0.28, *n* = 8; 20 Hz, 0.57 ± 0.30, *n* = 6. No significant differences were found. However, when the average amplitudes were compared across frequencies for only those PCs (*n* = 5) tested on all frequencies, the 20 Hz stimulation average (0.575 ± 0.34) was significantly higher than the averages for the other frequencies (0.5 Hz, 0.46 ± 0.28; 2 Hz, 0.38 ± 0.27; 5 Hz, 0.375 ± 0.32; 20 Hz vs.: 0.5 Hz, *p* = 0.019; 2 Hz, *p* = 0.039; 5 Hz, *p* = 0.0019). To investigate changes in alignment of the circular means between AC frequencies, the circular dispersion of the individual cell circular means at each frequency was calculated. The circular dispersion was lower for 20 Hz than the other frequencies, though the differences were not statistically significant because of the large SDs (0.5 Hz, 27.33 ± 54.50, *n* = 13; 2 Hz, 13.90 ± 20.33, *n* = 11; 5 Hz, 38.35 ± 55.79, *n* = 8; 20 Hz, 3.55 ± 4.39, *n* = 6, in all cases, *p* > 0.05). In sum, both a decrease in the phase variability between PC SS modulation and an increase in the amplitude of the individual cell modulation appear to contribute to the stronger population response at 20 Hz.

### Dependence of SS modulation on anatomical location of PC

Neuronal responses due to the direct effects of AC stimulation on the neuron itself depend on the relative orientations of the somatodendritic axis of the cell and the induced local electric field. In particular, if the field is directed somatopetally during the positive phase of the AC cycle, SS activity should modulate in phase with the AC stimulation, resulting in a circular mean with a phase angle close to 90°. If the field is oppositely directed (somatofugally), then an out-of-phase relationship is expected with a phase angle close to 270°, as was found for the majority of our PC population (Figures 4A–D).

However, some PCs had circular means with phase angles close to 90°, which raises the question of the extent to which the phase angle depends on a PC’s location, as a PC’s absolute orientation will vary with location along the folded cerebellar cortex. Moreover, since the somatodendritic axis of a PC always runs from the PC layer outward to the local surface of the molecular layer, we can use the PC’s location in each lobule to determine the relative orientation of its somatodendritic axis with respect to the stimulation electrode, which was located over the medial portion of lobule crus 1/vermal lobule 6. For example, a PC in the caudal wall of the paramedian lobule has its soma closer to the stimulation site, and thus has a somatodendritic orientation, whereas a PC in the rostral wall of the copula lobule has the opposite, dendrosomatic, orientation with respect to the apex of crus 1 (see Figure 1).

**FIGURE 6:**
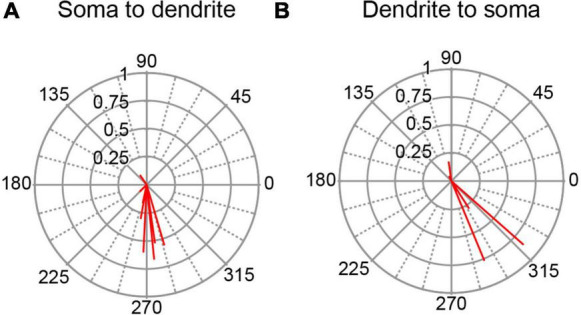
Dependence of SS modulation on orientation of PC soma-dendritic axis. **(A)** Polar plot of circular means for PCs with a somatodendritic orientation with respect to the AC stimulus electrode. **(B)** Same as panel **(A)** for PCs with a dendrosomatic orientation. AC stimulus frequency was 0.5 Hz.

All histologically-identified PCs with unimodal responses to AC stimulation were categorized according to their orientation, and the circular means were plotted for each PC group for the 0.5 Hz AC stimulation (Figure 6). PC responses in the somatodendritic group (*n* = 8) were quite homogeneous with almost all cells showing vectors pointing close to 270° (Figure 6A). The dendrosomatic group (*n* = 5) showed more spread and a slightly greater average phase angle, but nevertheless, phase angles between 180°–360° predominated in this group as well, particularly for the strongly modulated cells (Figure 6B). In sum, PC SS activity showed similar phase angles regardless of the orientation of the PC’s somatodendritic axis relative to the stimulus electrode location. That the phase of most PC circular means was between 180°–360° suggests that current is flowing outward through most of the cortex (not directly under the stimulus electrode) during the positive phase of the AC cycle and inward during its negative phase.

**FIGURE 7:**
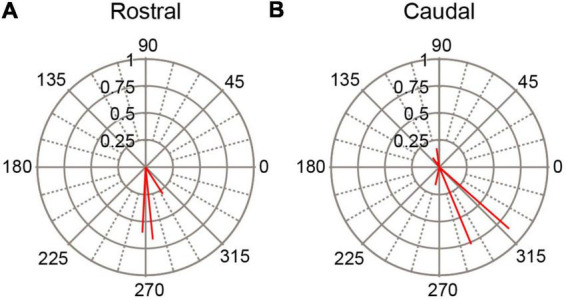
Variation of SS modulation with rostrocaudal location of PC. Polar plots of circular means for SS activity for **(A)** PCs located in lobules rostral to site of stimulation (crus1) and for **(B)** PCs located in lobules caudal to stimulation site. AC stimulus frequency was 0.5 Hz.

As a second way of investigating the dependence of SS responses on PC location we divided the PC population according to whether a cell was located rostral or caudal to crus 1. Thus, PCs in lobules 1–simplex were assigned to the rostral group, and those caudal to crus1 to the caudal group (PCs in crus 1 were excluded because the stimulation electrode was atop that lobule). The circular means for these two groups of PCs are shown in Figure 7. In both groups the predominant phase was between 180° and 360°, indicating SS activity modulated out-of-phase with the AC stimulus in both the rostral and caudal cerebellar cortex.

**FIGURE 8:**
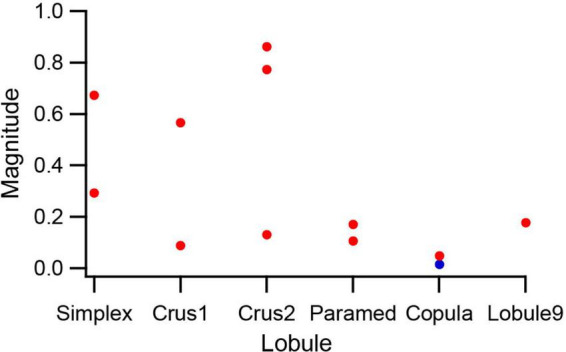
Lobular variation in SS modulation amplitude. PCs in which the same strength (500 μA) 0.5 Hz AC stimulus was tested were sorted according to the lobule that they were located in. The magnitude of the circular mean is plotted for each PC. Red circles indicate PCs with significant modulation (*p* < 0.05) and the blue circle indicates the one PC that had a non-significant modulation.

To examine the strength of modulation across the cerebellar cortex, PCs that were tested with 0.5 Hz, 500 μA AC stimulation (*n* = 12) were classified according to their lobule, and the magnitudes of their circular means were compared. Figure 8 plots the magnitudes for these PCs, arranged according to the rostrocaudal order of the lobules. PCs in the lobules farthest from the crus 1 showed relatively weak, but still significant, modulation (except for the one PC indicated by the blue circle). In contrast, for crus1 and its neighboring lobules both strong and weak modulation was observed. While the number of PCs in each lobule is low, the overall pattern suggests that the electric field generated by a localized stimulus site not unexpectedly decreases with distance, but that it still remains strong enough to significantly affect activity throughout much of the cerebellum. Possible explanations for the variation in modulation amplitude seen for crus1 and its neighboring lobules will be addressed in the discussion.

**FIGURE 9:**
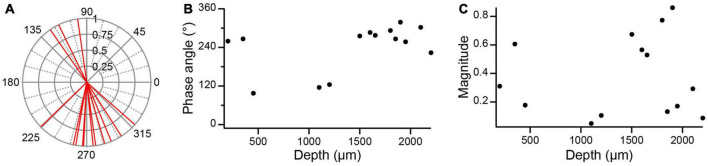
Dependence of SS modulation with lobular depth. **(A)** Polar plot of unit vectors showing the phase angles of all PC circular means to 0.5 Hz AC stimulation. **(B)** Phase angles plotted as a function of PC depth in lobule. Depths were estimated by measuring the distance between the plotted location of a PC (as determined from track reconstructions) and the surface of the lobule to which it was localized. **(C)** Plot of magnitude of circular means as a function PC depth in lobule.

The depth of the PC below the lobular apex is another potential parameter of its response to AC stimulation. Figure 9A shows the distribution of the phase angles for all PC SS responses to 0.5 Hz AC stimulation (*n* = 14, 1 PC with bipolar response was excluded). The phase angles and magnitudes of these responses are plotted as a function of lobular depth in Figures 9B, C, respectively. PCs at both superficial and deep locations had phase angles around 270°, indicating an out-of-phase modulation. At intermediate depths (∼500–1,300 μm) phase angles around ∼100° were observed suggesting a close to in-phase modulation for the three PCs recorded at these depths. However, the magnitude of the modulation of these PCs was relatively small (Figure 9C).

### Local PCs tend to modulate in-phase with AC stimulation

**FIGURE 10:**
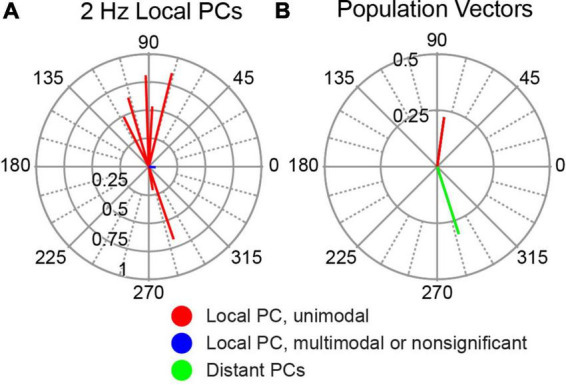
Modulation of SS activity of PCs located directly underneath stimulation site. **(A)** Polar plot of individual cell circular means of the 10 local PCs to 2 Hz AC stimulation. **(B)** Comparison of population vector of local PCs shown in panel **(A)** (red) with that of distant PCs (blue) redrawn from Figure 5A (2 Hz).

In our previous paper, PCs located just beneath the stimulus electrode were observed to modulate with AC stimulation (Asan et al., 2020). When tested with AC stimulation directed along the dorsoventral axis, similar to the stimulation arrangement used in the present study, the large majority of these cells showed a significant modulation (9/10), and most of those increased their SS activity during the positive phase of the AC cycle (6/9). To directly compare the activity of these local PCs to the distant PC population described above, we calculated the circular means for these PCs (Figure 10A). Most of these cells showed a strong modulation with phase angles close to 90°, with a few cells showing oppositely directed circular means. The population vectors for the local and distant PCs are compared in Figure 10B, which shows that the SS activity of the two PC populations modulated ∼180° out-of-phase with each other.

### AC stimulation modulates CN spiking

Recordings were obtained from 20 neurons within the CN. Eighteen were histologically-localized (medial, 4; anterior interpositus, 2; posterior interpositus, 10; lateral, 2). The remaining two cells were categorized as CN neurons based on the micrometer depth reading (∼3,550–4,000 μm below the surface) and presumed track trajectory. CN neurons displayed spontaneous firing rates of 27.52 ± 24.46 Hz with patterns that ranged from relatively tonic to bursting (Figure 2K), as previously described (Blenkinsop and Lang, 2011). AC stimulation was effective in modulating the activity of 17/20 CN cells at one or more stimulus frequencies (Figures 2D–F). Of those 17 cells, almost all showed a unimodal response to some or all stimulation frequencies (16/17 cells), with only 6 cells showing a bimodal response pattern to one or more stimulus frequencies. Moreover, most modulated cells (13/17) showed a significant modulation to all tested frequencies. However, the magnitudes of the circular means for individual CN cells tended to be smaller than those found for PCs, despite using equal or higher AC intensities, although the difference in the average amplitude of the individual cell circular means was only significant for 20 Hz stimulation (*p* = 0.035) (Figures 4E–H). A similar difference was found when comparing the modulation of cells during identical strength stimulation (500 μA) (data not shown). Correspondingly, the CN population vectors were smaller than PC SS population vectors (Figures 5A, B).

Like PC SS activity, the activity of most CN cells showed an out-of-phase relationship with the AC stimulation cycle, which was reflected by their circular means having phase angles between 180° and 360° (Figures 4E–H) and by similarly directed population vectors (Figure 5B). However, unlike for PC SS activity, the magnitude of the CN population vector dropped with increasing frequency. This frequency dependence appeared to reflect changes in the modulation amplitude of individual cells, as the average magnitudes of the individual cell circular means decreased steadily with frequency (0.5 Hz, 0.301; 2 Hz, 0.288; 5 Hz, 0.226; 20 Hz, 0.209); however, the correlation with frequency was not significant (*r* = −0.82, *p* = 0.18). In addition, the circular dispersion of the individual cell activity increased steadily with frequency. In sum, the changes in the magnitudes and the spread of the phase angles of the individual cell circular means with frequency both may underlie the reduction of the CN population vector with increasing stimulus frequency.

### Vestibular nuclear cell responses

A total of 11 neurons were histologically-localized to one of the VN (medial, *n* = 7; lateral, *n* = 2; superior, *n* = 2). The spontaneous firing rates were 15.48 ± 12.79 Hz (Figure 2L). Individual VN cells often could be strongly modulated (Figures 2G–I), as indicated by the amplitudes of their individual cell circular means (Figures 4I–L) and population vectors (Figure 5C) at each stimulus frequency. The phase angles showed that for all frequencies except 20 Hz, most VN cell activity modulated out of phase with the AC stimulus, but that the activity of some cells modulated in phase (out/ in/ non-unimodal; 0.5 Hz: 8/ 2/ 1; 2 Hz: 6/ 3/ 1; 5 Hz: 4/ 2/ 2; 20 Hz: 1/ 2/ 2).

### Cerebellar cortical cells show weaker modulation than PC population

**FIGURE 11:**
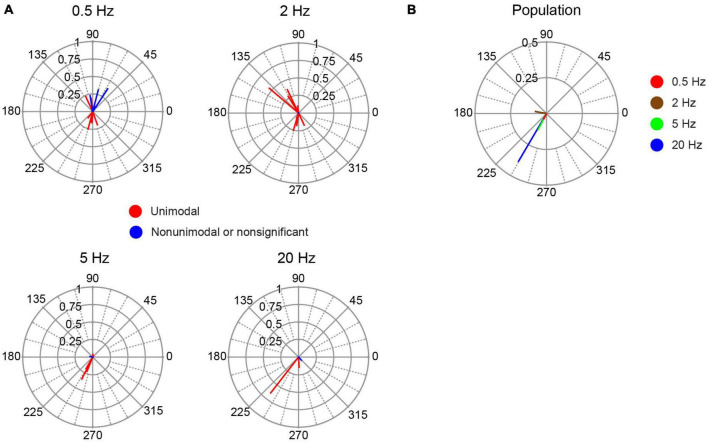
Phase distribution of responses of non-identified cortical cells to AC stimulation. **(A)** Plots of the individual cell circular means during AC stimulation at the frequency indicated above each plot. **(B)** Plot of population vectors for each frequency.

Fifteen cells could be localized to cerebellar cortical locations by micrometer readings or histological localization (Figure 1, light blue circles). These cells likely include various cortical interneurons, though cases of PC SS activity cannot be absolutely ruled out. The response of this group of cells to AC stimulation was more diverse and generally weaker than identified PC SS activity. The average amplitude of the individual cell circular means for non-identified cortical neurons was smaller than identified PC SS (Figure 11). An ANOVA (cell group X stimulus frequency X stimulus intensity) showed only a main effect of cell group (*p* = 0.001). In addition, the phase angles of the vectors were more widely distributed to show both in-phase and out-of-phase relationships at the lower frequencies (Figure 11A). As a result, the population vectors are quite small at all frequencies except 20 Hz (Figure 11B); however, only two of four cells tested at that frequency showed significant modulation.

### Measurement of electric field in cerebellum

**FIGURE 12:**
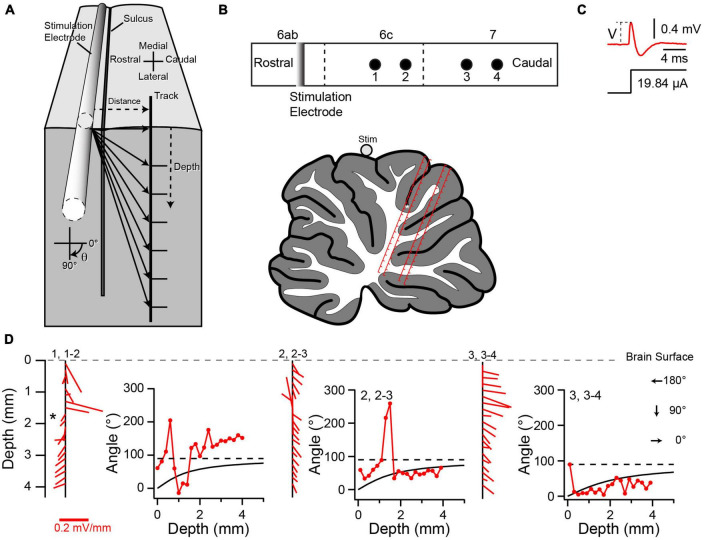
Variation in local electric field in cerebellum. **(A)** Schematic of experimental set up for field recordings. Stimulation electrode (silver wire) is placed on brain surface and aligned with the mediolateral axis. Tracks are made perpendicular to the local surface. Solid lines with arrows indicate the directions of the electric field in the parasagittal plane containing the recording track at the recording sites indicated by the tips of the arrows for a homogeneous medium. **(B)** Top schematic is a surface view that shows the relative locations of the stimulation electrode and four tracks (black circles). Tracks 1 and 2, and 3 and 4, are spaced by 254 μm. Tracks 2 and 3 are spaced by 508 μm along the surface. Vermal lobules are indicated above the rectangle. Vertical dashed lines indicate sulci. Lower schematic was traced from the histological section containing the tracks. The locations of the tracks (red lines) are superimposed on the schematic. Each track is 4 mm long, and the tick marks on the tracks refer to the depths at which recordings were made. Note that depths don’t account for shrinkage due to histological processing, and so are approximate. The white asterisk refers to the location where a robust change in the electrical field direction was observed. **(C)** Average voltage response (top trace) to five current pulses (bottom trace). V indicates the peak voltage that was used to compute the electric field. **(D)** Variation of the electric field along recording tracks. Each vertical black line with red line segments extending from it shows the variation of the electric field. The red lines are the calculated electric field vectors. The vertical position of the starting point of each vector (at the vertical black line) indicates the depth below the surface for which the field was determined, and is plotted midway between the two recording points along the track that was used to calculate the dorsoventral component of the vector. This track is indicated by the first number above the black line. The following pair of numbers indicates the two neighboring tracks used to calculate the rostrocaudal component. The experimentally-determined angle θ of the electric field (as calculated from the dorsoventral and rostrocaudal components) is plotted as a function of depth to the right of the corresponding electric field vectors. The black curve in each plot shows the predicted value of θ as a function of depth for a homogeneous medium. Dashed line indicates the 90° asymptote of the theoretical curve. The location of the asterisk in 1, 1–2 corresponds to the one in the schematic of panel **(B)**.

**FIGURE 13:**
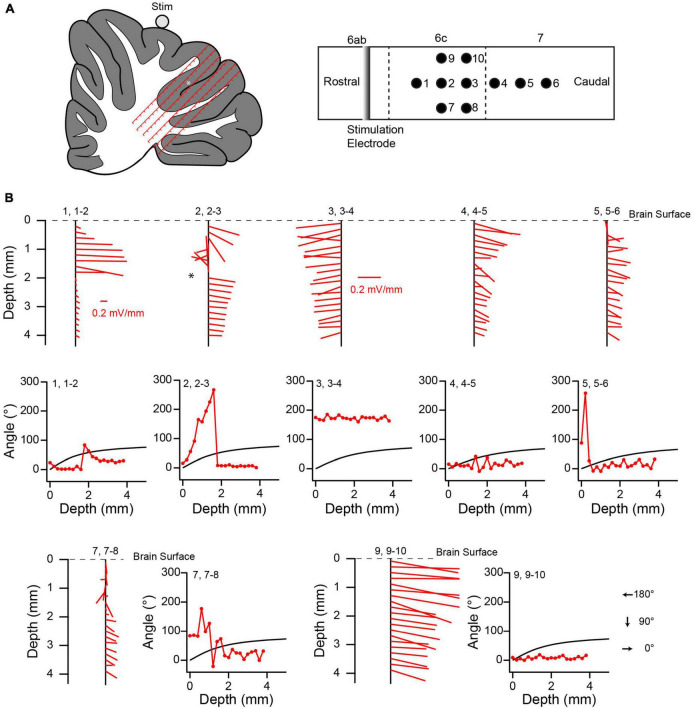
Local electric field distribution in another experiment. **(A)** Schematics showing relative positions of stimulus electrode and recording tracks. **(B)** Variation of electric field along recording tracks. Same naming conventions as Figure 12. Scale in panel **(B)**, track 1, 1–2 also applies to 2, 2–3. The scale in 3, 3–4 applies to all other tracks.

The responses of PC SSs and CN cells across the cerebellum suggest that the electric field induced by the AC stimulation differs from what would be expected in a homogenous resistive medium (i.e., a field that is always directed from the current source to the recording site and that decreases with increasing separation of the source and recording site). To investigate the structure of the electric field in the cerebellum, positive current pulses (∼20–40 μA, 1 s) were applied using a silver wire electrode placed on the surface of the vermis and oriented mediolaterally (lobule 6 or 7) (*n* = 2 animals). Responses to these stimulus pulses were recorded at 200 μm intervals along tracks made perpendicular to the lobular surface to depths of 3–4 mm (Figures 12A, B, 13A). Typically, the amplitude of the evoked voltage response (V) was determined from the average response to a series of 5–10 current pulses (Figure 12C), and used to calculate the electric field (see Methods). Tracks entered the lobules caudal to the one on which the stimulation electrode was placed, and were located in a parasagittal plane that crossed the stimulation electrode near its midpoint to minimize edge effects due to the finite length of the stimulation electrode (Figures 12B, 13A).

The distribution of voltage responses in both experiments showed that the current stimuli induced complex, asymmetric fields. The electric field component in the parasagittal plane at various locations in the cerebellum is shown in Figures 12D, 13B as red lines extending from vertical black lines. The red lines are vectors representing the electric field magnitude and direction at different points in the cerebellum, and the starting point of each vector on the black vertical line represents the location of the field. If a positive current pulse induced a symmetric, outwardly-directed field, as expected for a homogeneous resistive medium, the vectors should decrease in magnitude with depth, because of the increasing distance between the stimulus and recording electrodes. In contrast, the calculated magnitude of the electric field in the parasagittal plane (indicated by the lengths of the red lines) did not decrease monotonically with depth. Rather, although a trend to smaller amplitudes can often be seen, there are multiple exceptions where the vector lengths increased.

Even more striking is the deviation in direction of the field from what would be predicted for a homogeneous resistive medium. Given that the tracks were all located caudal to the stimulation electrode, the field should always be oriented caudally, and more specifically, should, in the parasagittal plane, point radially away from the stimulation electrode to the recording site. That is, θ should increase from 0° to 90°as one proceeds ventrally from the dorsal surface (Figure 12A). In contrast, although plots of the calculated direction of the field as a function of depth show that the field is caudally-directed in many cases, there are multiple exceptions in which a rostrally-directed field was found (i.e., θ between 90° and 270°, leftward pointing vectors in Figures 12D, 13B, plots of the field direction versus depth). Moreover, while in some cases (e.g., Figure 12D, track 2, 2–3) the measured field direction could match the predicted angle for at least a portion of the track, in the large majority of cases very little correspondence was observed, with measured values deviating in both directions from the predicted ones.

It is worth noting that some depths were associated with a sudden reversal in field direction along the rostrocaudal axis (i.e., angles going from <90° to >90° and vice versa) (Figure 12C, asterisk). Histological analysis suggests that these sometimes occurred where the track of at least one of the electrodes appeared to be crossing a sulcus from one lobule to the next. However, further analysis is needed to verify this relationship, particularly as this was not always observed when tracks must have crossed between lobules.

## Discussion

The present paper represents the first relatively global description of cerebellar neuronal responses to applied AC stimulation under *in vivo* conditions, and builds upon our previous work describing the responses of PCs located at the stimulation site (Asan et al., 2020). The results indicate that AC stimulation applied to a local area on the cerebellar surface can lead to widespread, largely opposing modulation of SS activity between PCs located close to and far from the stimulation site, and in addition, can cause modulation of CN and VN neurons. The pattern of the SS responses in particular suggests that the electric field induced in the cerebellum by AC stimulation differs significantly from a radially-directed field that would occur in a homogeneous and isotropic medium. Direct measurements of the electric field at various distances from the electrode confirmed the non-radial nature of the field.

### PC SS response patterns across the cerebellar cortex suggest a non-radial pattern of current flow

It has been shown in multiple cell types, including PCs, that an imposed external electric field of sufficient magnitude that is directed from the dendrites toward the soma will depolarize the soma and increase spiking (Purpura and McMurtry, 1965; Ranck, 1975; Chan and Nicholson, 1986; Chan et al., 1988; Bikson et al., 2004; Rahman et al., 2013). An electric field may also influence the activity of a cell indirectly, via modulation of other cells that synapse onto the cell in question. However, earlier work suggests that when an AC stimulus is applied to the cerebellar surface *in vitro* at least, the direct effects dominate in determining the PC responses (Chan and Nicholson, 1986). Our results provide support for this to be the case *in vivo* as well, as detailed below.

If we then assume that PC SS modulation is primarily due to a direct polarization of the PC membrane by the AC stimulation, the modulation patterns we observed can be used to infer the direction of the local electric field throughout the cerebellum, given that PCs have a dendritic tree that is orthogonal to the local cerebellar surface with a soma located deep to the dendritic tree. Specifically, when PC SS activity modulates in-phase with the AC stimulation, it implies that the local electrical field is directed inward from the local surface during the positive AC phase and outward during the negative phase, and vice versa, when PC SS activity modulates out-of-phase with respect to AC stimulation.

The predominant SS modulation pattern we observed can be summarized as follows: in most local PCs near the site of stimulation, SS activity modulated in-phase with the AC stimulation, whereas SSs of most PCs located elsewhere modulated out-of-phase. Moreover, the modulation was strongest for cerebellar regions close to the stimulation site, but remained significant (albeit weaker) at quite distant sites.

The in-phase relationship observed for local PCs indicates that current (and the electric field) is directed inward (from pial surface through the local cortex) during the positive phase of the AC cycle and outward during its negative phase, which is not unexpected, and agrees with what was shown in the *in vitro* turtle preparation (Chan and Nicholson, 1986; Chan et al., 1988). The out-of-phase relationship to the AC stimulation typically seen for non-local PCs suggests that current is flowing outward through most of rest of the cerebellar cortex that is not in the immediate vicinity (same lobular apex) of the electrode. Thus, current enters through the local surface being stimulated, flows within the cerebellar mass, and then out through the more distant cortex. That nearly all non-local PCs showed the out-of-phase relationship argues against two other possible patterns of current flow that might plausibly have been expected.

One of these possibilities is that current flows radially from the stimulation electrode, i.e., that the sulci and the anisotropies and inhomogeneities of the cerebellar tissue do not produce major changes in the overall electric field. With an approximately radial pattern of current flow, one would expect to observe significant numbers of both in-phase and out-of-phase modulation of PC SS in non-local PCs, as the phase of the modulation should depend on the orientation of the PC somatodendritic axis relative to the stimulation electrode. In particular, the modulation of PCs on opposite walls of a lobule (or opposite sides of a sulcus) should have had a 180° phase relationship, which was not observed.

Another possible pattern is for current to flow primarily around the exterior of the cerebellar cortex and down into the sulci, before flowing inwardly at all points. This pattern would produce in-phase PC SS modulation at all locations, regardless of PC orientation. Again, our data do not support this possibility, as such a pattern would lead to all PC SS activity showing an in-phase relationship to the AC stimulation, almost the opposite of what was observed.

One limitation of our current database is that most of our PCs were located on the walls of lobules. So, we do not have a large sample of PCs located on the apices of non-stimulated lobules. It is possible that they could show an in-phase relationship on some (e.g., lobules adjacent to the stimulated lobule) or all lobules, implying a current path that goes around the exposed exterior surface of the cerebellum, and possibly into the outer portion of the sulci before entering the cerebellar cortex itself. Nevertheless, our results, at a minimum, suggest that this pattern would be limited to the superficial aspect of the lobules.

### PC SS modulation is likely due to a direct polarization of the PC

The above inferences about local current flow throughout the cortex from PC SS modulation phase assume that AC stimulation acted mainly via directly polarizing PCs. To strengthen this conclusion, it is necessary to consider whether synaptic effects played a role in the SS response patterns. Indeed, stellate cell activity was also found to be modulated by AC fields in an *in vitro* turtle preparation, raising the possibility that they could affect PC responses to AC stimulation; however, the modulation was found to be generally weaker, and show more variability in its phase relationship to the stimulus cycle, both of which would lower its efficacy in driving PC SS activity (Chan and Nicholson, 1986).

Our unidentified cortex population comprises a mix of cerebellar cortical interneurons (though we cannot absolutely exclude the presence of PC activity). At the lower stimulus frequencies, the vectors were generally small, consistent with the prior *in vitro* findings. Moreover, if SS modulation were being driven by interneuron activity, the two populations would be expected to have an out-of-phase relationship, given the inhibitory nature of stellate cells. Indeed, this would be true for most cerebellar interneurons. In contrast, our population had phase angles both less and greater than 180°, which combined with the generally weaker amplitude of modulation, resulted in population vectors that were small in amplitude. Thus, the interneuron population activity would not likely provide a strong, properly timed signal for driving SS activity. Moreover, at the higher stimulus frequencies (5 Hz and 20 Hz), the interneuron vectors were directed downward (>180°), indicating that their activity modulated in-phase with SS activity, and so could not be the prime driver of SS modulation. In contrast, if anything, interneurons may be acting to tamp down the PC SS modulation; however, the strength of this effect remains to be investigated.

### CN and VN response patterns in relation to PC SS patterns

The activity of both CN and VN cells was significantly modulated by AC stimulation. The CN are located ∼3–5 mm below the cerebellar surface, and the VN are located at an even greater distance. At such distances one might expect the electric field to be too small to drive neuronal activity; however, PCs located at similarly large distances had modulated SS activity, and our field measurements show that the field amplitude can remain significant at such distances. Thus, CN and VN modulation could at least partly be due to direct polarization of these nuclear neurons by the electric field.

It would seem likely, given how strongly PC SS activity was modulated, that CN and VN modulation is also due to indirect synaptic effects. However, the relationship is not straightforward, as the inhibitory nature of PCs would predict an out-of-phase relationship of PCs with both CN and VN activity. In addition, one would also expect predictable increases in the phase angle with frequency, because the fixed conduction time of the PC to CN or VN projection would translate to an increasing phase delay with increasing frequency; however, this effect would be expected to be small for the tested frequencies, given the short conduction time of the PC axon, and thus may have been obscured by the cell-to-cell variability in phase. In contrast to the predicted out-of-phase relationship, most PCs, CN, and VN cells modulated in-phase with each other (and out-of-phase with the AC stimulation). Possibly the PCs local to the stimulation site, which predominantly modulate in-phase with the AC stimulation, are driving CN and VN activity, and the more widespread distant PC population simply acts to tamp down the effect of the local PCs. However, the stimulation site was relatively constant across experiments, whereas cells were recorded from multiple widespread regions of the CN and VN, which would not all receive synaptic input from the same local cell population. Furthermore, the strength of the CN modulation decreased with AC frequency, whereas that of PC SS activity increased. This relationship is more consistent with PC SS activity acting to tamp down the CN modulation rather than driving it, at least under the present experimental conditions. In sum, it is hard to disentangle the direct and indirect effects of the AC stimulation on CN and VN cells, but it seems likely that the response of these cells reflects the combination of both direct and indirect effects.

### AC stimulation produces a non-radial electric field in the cerebellum

In these experiments we used two stimulation electrode configurations, a coil that forms a disk-like contact with cerebellar surface, and a straight wire. The electric field in a homogenous and isotropic volume conductor, due to a current injected through a disk electrode on the surface, decreases as a function of the vertical distance (z) into the volume according to the following formula: |E| ∝ 1 / (z^2^ + a^2^), where a is the radius of the electrode (1.5 mm in our case) and |E| is the magnitude of the field (Wiley and Webster, 1982; Asan et al., 2019). This analytical equation predicts that the field is more or less uniform for z < a, and decreases monotonically following the 1/z^2^ curve for z >> a. Similarly, the theoretical field generated by a straight wire electrode in a homogeneous medium can be approximated as a finite linear current source of length L, with |E| ∝ 1/ (z*((L/2)^2^ + z^2^)^1/2^), where z is the perpendicular distance from the wire. Again, |E| continually decreases with increasing z, and follows the 1/z^2^ curve when z >> L. While the stimulus electrodes that were used are only approximations of an ideal disk and line, the experimentally-determined electric fields deviate significantly from the theoretically predicted fields in ways that are unlikely to be due to these approximations. Specifically, it is hard to explain the observed increases in field strength with distance, and rostrally-directed fields at certain depths, rather than the field direction asymptotically approaching a dorsoventral direction from a caudal direction.

The deviations from the theoretical fields suggest that the cerebellum is not a homogenous and isotropic volume conductor. Indeed, this is consistent with measurements of cerebellar conductivity, which show that the cerebellum is anisotropic (Yedlin et al., 1974; Nicholson and Freeman, 1975; Okada et al., 1994). Such anisotropies may be due to a higher conductivity along fiber tracts, which could substantially slow down the decrease in the electric field by depth. Moreover, this may explain the somewhat surprisingly strong modulation of CN neurons and distant PC SS activity we observed. In contrast, if the cerebellar tissue were homogeneous and isotropic, nuclear cells would experience several times smaller electric fields than the PCs near the electrode as estimated from the relationship for a disk electrode given above. That is, PCs immediately underneath the electrode (z ≅ 250 μm) would experience 4.86 times larger electric fields than the nuclear cells at a depth of 3 mm from the pia and 7.89 times larger from those at 4 mm. In contrast, CN modulation was of the same order of magnitude (albeit slightly weaker on average) as PCs, which is consistent with the less than predicted decline of the electric field that was observed.

Additional contributions to the anisotropy of the cerebellum may come from the pial covering that follows the folding of the cortex, if it acts as a barrier to translobular current flow. These and other factors likely route current to the deeper cerebellar regions, and may explain the widespread and out-of-phase firing pattern observed for PCs located at a distance from the stimulation electrode.

In conclusion, our results suggest that focal AC stimulation produces modulation of PCs throughout much of the cerebellar cortex, and cells of the CN and VN. The pattern of modulation we observed has implications for cerebellar transcranial stimulation, and in particular, suggests that assuming radial spread of current from a surface electrode may not be valid. However, there are several important caveats for directly translating our results to transcranial stimulation as applied in humans. First, interposition of the skull, and the skin, in particular, will attenuate the current entering the cerebellum. Indeed, measurements of the electric field induced in the rat cerebrum showed that electrode placement on the skull or skin results in attenuation of field relative to that induced by an electrode on the dural surface, particularly for latter case (Asan et al., 2019). Second, the interposition of the skull and skin between the electrode and brain in transcranial stimulation will cause the current to spread more widely before entering the cerebellar surface than in our experimental arrangement. Here, the overlying structures were removed and stimulation was applied directly to the cerebellar surface in order to minimize the variations between the animals and to have better localization of the current entry point into the cerebellum. Current spread across these non-CNS structures in the case of transcranial stimulation would likely lead to more orthogonal field orientations on the cerebellar surface (i.e., a wider current entry region); however, we predict that the results for the cells deep inside the cerebellar folds we observed with direct stimulation of the cerebellar surface will generalize to the transcranial case.

In addition, the larger size of the human cerebellum needs to be taken into account. The greater depth of the CN and VN in humans suggests that they would likely experience a more attenuated electric field, and thus, the balance of the mechanisms causing CN and VN modulation (direct versus modulation of PC activity) will likely be shifted to modulation of PC activity. However, our results raise the possibility that direct effects may occur at depths significantly greater than expected if the cerebellum were an isotropic and homogeneous medium. Specifically, the deviations from predicted changes in the magnitude and orientation of the electric field with depth suggest that the presence of high conductivity pathways that would direct current to deeper regions, allowing a direct effect on cells in these regions. Such pathways are likely present in the human cerebellum as well, and thus our results imply such high conductivity pathways may be an important factor in the clinical setting.

In conclusion, understanding how the cellular effects of the electric fields vary depending on the anatomical features will help design electrode geometries that can better target the desired groups of cells and produce the desired therapeutic effects.

## Data availability statement

The raw data supporting the conclusions of this article will be made available by the authors, without undue reservation.

## Ethics statement

This animal study was reviewed and approved by the Institutional Animal Care and Use Committees of New York University Grossman School of Medicine and Rutgers University.

## Author contributions

EL, MS, and BA contributed to the conception and design of the study. BA, EL, EC, and AA performed the experiments and collected the data. BA, EL, EC, RR, and SV analyzed the data. EL wrote the first draft of the manuscript. MS wrote sections of the manuscript. All authors contributed to manuscript revision, read, and approved the submitted version.

## References

[B1] Asan A. S. Gok S. Sahin M. (2019). Electrical fields induced inside the rat brain with skin, skull, and dural placements of the current injection electrode. *PLoS One* 14:e0203727. 10.1371/journal.pone.0203727 30629578 PMC6328113

[B2] Asan A. S. Lang E. J. Sahin M. (2020). Entrainment of cerebellar purkinje cells with directional AC electric fields in anesthetized rats. *Brain Stimul.* 13 1548–1558. 10.1016/j.brs.2020.08.017 32919090 PMC7722055

[B3] Bikson M. Inoue M. Akiyama H. Deans J. K. Fox J. E. Miyakawa H. (2004). Effects of uniform extracellular DC electric fields on excitability in rat hippocampal slices in vitro. *J. Physiol.* 557 175–190. 10.1113/jphysiol.2003.055772 14978199 PMC1665051

[B4] Blenkinsop T. A. Lang E. J. (2011). Synaptic action of the olivocerebellar system on cerebellar nuclear spike activity. *J. Neurosci.* 31 14708–14720. 10.1523/JNEUROSCI.3323-11.2011 21994387 PMC3711508

[B5] Chan C. Y. Nicholson C. (1986). Modulation by applied electric fields of Purkinje and stellate cell activity in the isolated turtle cerebellum. *J Physiol* 371 89–114. 10.1113/jphysiol.1986.sp015963 3701658 PMC1192712

[B6] Chan C. Y. Hounsgaard J. Nicholson C. (1988). Effects of electric fields on transmembrane potential and excitability of turtle cerebellar Purkinje cells in vitro. *J. Physiol.* 402 751–771. 10.1113/jphysiol.1988.sp017232 3236254 PMC1191919

[B7] Darch H. T. Cerminara N. L. Gilchrist I. D. Apps R. (2018). “Non-invasive stimulation of the cerebellum in health and disease,” in *Transcranial magnetic stimulation in neuropsychiatry*, ed. Ustohal L. (London: IntechOpen), 23–40. 10.5772/intechopen.73218

[B8] Ferrucci R. Giannicola G. Rosa M. Fumagalli M. Boggio P. S. Hallett M. (2012). Cerebellum and processing of negative facial emotions: Cerebellar transcranial DC stimulation specifically enhances the emotional recognition of facial anger and sadness. *Cogn Emot* 26 786–799. 10.1080/02699931.2011.619520 22077643 PMC4234053

[B9] Lang E. J. (2018). “Multielectrode arrays for recording complex spike activity,” in *Extracellular recording approaches, neuromethods*, ed. Sillitoe R. V. (Berlin: Springer). 10.1007/978-1-4939-7549-5_4

[B10] Naro A. Bramanti A. Leo A. Manuli A. Sciarrone F. Russo M. (2017a). Effects of cerebellar transcranial alternating current stimulation on motor cortex excitability and motor function. *Brain Struct. Funct.* 222 2891–2906. 10.1007/s00429-016-1355-1 28064346

[B11] Naro A. Milardi D. Cacciola A. Russo M. Sciarrone F. La Rosa G. (2017b). What do we know about the influence of the cerebellum on walking ability? Promising findings from transcranial alternating current stimulation. *Cerebellum* 16 859–867. 10.1007/s12311-017-0859-4 28456901

[B12] Nicholson C. Freeman J. A. (1975). Theory of current source-density analysis and determination of conductivity tensor for anuran cerebellum. *J. Neurophysiol.* 38 356–368. 10.1152/jn.1975.38.2.356 805215

[B13] Okada Y. C. Huang J. C. Rice M. E. Tranchina D. Nicholson C. (1994). Origin of the apparent tissue conductivity in the molecular and granular layers of the in vitro turtle cerebellum and the interpretation of current source-density analysis. *J. Neurophysiol.* 72 742–753. 10.1152/jn.1994.72.2.742 7983532

[B14] Purpura D. P. McMurtry J. G. (1965). Intracellular activities and evoked potential changes during polarization of motor cortex. *J. Neurophysiol.* 28 166–185. 14244793 10.1152/jn.1965.28.1.166

[B15] Rahman A. Reato D. Arlotti M. Gasca F. Datta A. Parra L. C. (2013). Cellular effects of acute direct current stimulation: Somatic and synaptic terminal effects. *J. Physiol.* 591 2563–2578. 10.1113/jphysiol.2012.247171 23478132 PMC3678043

[B16] Ranck J. B. Jr. (1975). Which elements are excited in electrical stimulation of mammalian central nervous system: A review. *Brain Res.* 98 417–440. 1102064 10.1016/0006-8993(75)90364-9

[B17] Wiley J. D. Webster J. G. (1982). Analysis and control of the current distribution under circular dispersive electrodes. *IEEE Trans. Biomed. Eng.* 29 381–385. 10.1109/TBME.1982.324910 7084970

[B18] Yedlin M. Kwan H. Murphy J. T. Nguyen-Huu H. Wong Y. C. (1974). Electrical conductivity in cat cerebellar cortex. *Exp. Neurol.* 43 555–569. 10.1016/0014-4886(74)90195-2 4827164

[B19] Zar J. H. (1999). *Biostatistical analysis*, 4th Edn. London: Pearson Education.

